# Supplementary material for: Can additional funding improve mental health outcomes? Evidence from a synthetic control analysis of California’s millionaire tax
Source: PLoS One. 2022 Jul 27;17(7):e0271063. doi: 10.1371/journal.pone.0271063 (PMC9328510; doi:10.1371/journal.pone.0271063)
Supplement: S1 Table — (DOCX) [file pone.0271063.s001.docx]

| **S1 Table.** **Donor States and Weights for Synthetic Control Analysis of California’s General Population.** | |
| --- | --- |
| State | Weight |
| Connecticut | 0.062 |
| Delaware | 0.040 |
| Georgia | 0.054 |
| Hawaii | 0.049 |
| Iowa | 0.050 |
| Illinois | 0.062 |
| Massachusetts | 0.043 |
| Maryland | 0.053 |
| Michigan | 0.038 |
| Minnesota | 0.046 |
| New Hampshire | 0.113 |
| New Jersey | 0.050 |
| New York | 0.077 |
| Ohio | 0.051 |
| Pennsylvania | 0.060 |
| Rhode Island | 0.047 |
| Texas | 0.054 |
| Virginia | 0.053 |
