# Supplementary material for: Can additional funding improve mental health outcomes? Evidence from a synthetic control analysis of California’s millionaire tax
Source: PLoS One. 2022 Jul 27;17(7):e0271063. doi: 10.1371/journal.pone.0271063 (PMC9328510; doi:10.1371/journal.pone.0271063)
Supplement: S2 Table — (DOCX) [file pone.0271063.s002.docx]

| **S2 Table. Earmarked Mental Health Tax Effect on Suicide Deaths Among California’s General Population, 2012-2019.** | | | |
| --- | --- | --- | --- |
| Year | Treatment effect (Reduction in deaths per 100,000 adults) | Population (millions) | Equivalent effect  (Reduction in  suicide deaths) |
| 2012 | -1.30 | 38.0 | 494 |
| 2013 | -1.10 | 38.3 | 421 |
| 2014 | -1.50 | 38.6 | 579 |
| 2015 | -1.80 | 38.9 | 700 |
| 2016 | -1.71 | 39.2 | 670 |
| 2017 | -2.48 | 39.4 | 977 |
| 2018 | -2.09 | 39.5 | 826 |
| 2019 | -2.20 | 39.5 | 869 |
| Total |  |  | 5,536 |
| The treatment effect was non-significant prior to 2012; some estimates may differ due to rounding; adult population numbers drawn from United States Census Bureau QuickFacts. | | | |
