# Supplementary material for: Can additional funding improve mental health outcomes? Evidence from a synthetic control analysis of California’s millionaire tax
Source: PLoS One. 2022 Jul 27;17(7):e0271063. doi: 10.1371/journal.pone.0271063 (PMC9328510; doi:10.1371/journal.pone.0271063)
Supplement: S3 Table — (DOCX) [file pone.0271063.s003.docx]

| **S3 Table. Donor Pool States and Weights for Sex and Race Analyses.** | | | | | |
| --- | --- | --- | --- | --- | --- |
| Males | Females | Whites | Blacks | Hispanics | Asians |
| Connecticut (0.058)  Delaware (0.043)  Hawaii (0.050)  Iowa (0.051)  Illinois (0.060)  Massachusetts (0.045)  Maryland (0.053)  Michigan (0.041)  Minnesota (0.048)  Nebraska (0.076)  New Hampshire (0.087)  New Jersey (0.05)  New York (0.074)  Ohio (0.051)  Pennsylvania (0.058)  Rhode Island (0.049)  Texas (0.053)  Virginia (0.054) | Alabama (0.037)  Georgia (0.040)  Iowa (0.085)  Indiana (0.049)  Kansas (0.036)  Kentucky (0.036)  Louisiana (0.044)  Maine (0.018)  Michigan (0.059)  Mississippi (0.049)  North Carolina (0.020)  Nebraska (0.245)  New Hampshire (0.029)  Ohio (0.080)  Pennsylvania (0.072)  Texas (0.034)  Wisconsin (0.033)  West Virginia (0.034) | Connecticut (0.040)  Delaware (0.116)  Illinois (0.045)  Indiana (0.075)  Massachusetts (0.034)  Maryland (0.052)  Michigan (0.057)  Minnesota (0.046)  Nebraska (0.062)  New Hampshire (0.066  New Jersey (0.034)  New York (0.034)  Ohio (0.053)  Pennsylvania (0.058)  Rhode Island (0.039)  Texas (0.069)  Wisconsin (0.064) | Alabama (0.054)  Arkansas (0.055)  Arizona (0.057)  Georgia (0.053)  Indiana (0.059)  Kentucky (0.060)  Louisiana (0.053)  Michigan (0.056)  Mississippi (0.053)  North Carolina (0.054)  New Jersey (0.051)  Ohio (0.056)  Oklahoma (0.065)  Pennsylvania (0.058)  South Carolina (0.052)  Tennessee (0.054)  Texas (0.053)  Virginia (0.055) | Arizona (0.016)  Colorado (0.013)  Connecticut (0.026)  Florida (0.018)  Georgia (0.009)  Illinois (0.049)  Massachusetts (0.042)  Michigan (0.015)  North Carolina (0.063)  New Jersey (0.526)  New Mexico (0.004)  Nevada (0.015)  New York (0.135)  Pennsylvania (0.02)  Texas (0.027)  Washington (0.025) | Florida (0.133)  Hawaii (0.084)  Illinois (0.161)  New Jersey (0.192)  New York (0.168)  Texas (0.154)  Washington (0.108) |

The donor pools vary in size due to data limitations. For some groups, there are very few states with mortality comparable to California. To ensure a balanced donor pool—i.e., one with an equivalent number of states with mortality above and below California—other non-comparable states were not added simply to enlarge the pool.
