# Supplementary material for: Can additional funding improve mental health outcomes? Evidence from a synthetic control analysis of California’s millionaire tax
Source: PLoS One. 2022 Jul 27;17(7):e0271063. doi: 10.1371/journal.pone.0271063 (PMC9328510; doi:10.1371/journal.pone.0271063)
Supplement: S4 Table — (DOCX) [file pone.0271063.s004.docx]

| **S4 Table. Donor Pool States and Weights for Age Group Analyses.** | | | | | |
| --- | --- | --- | --- | --- | --- |
| 15-24 | 25-34 | 35-44 | 45-54 | 55-64 | 65+ |
| Connecticut (0.124)  Florida (0.067)  Illinois (0.090)  Massachusetts (0.203)  Michigan (0.055)  New Jersey (0.302)  New York (0.16) | Connecticut (0.101)  Georgia (0.072)  Hawaii (0.047)  Illinois (0.092)  Maryland (0.096)  Massachusetts (0.119)  New Jersey (0.129)  New York (0.195)  Texas (0.076)  Virginia (0.071) | Connecticut (0.087)  Georgia (0.056)  Hawaii (0.053)  Illinois (0.071)  Maryland (0.086)  Massachusetts (0.097)  Minnesota (0.061)  Nebraska (0.044)  New Hampshire (0.047)  New Jersey (0.125)  New York (0.173)  Ohio (0.052)  Virginia (0.047) | Arkansas (0.043)  Connecticut (0.054)  Georgia (0.049)  Hawaii (0.047)  Idaho (0.043)  Illinois (0.057)  Kansas (0.044)  Louisiana (0.051)  Maryland (0.058)  Massachusetts (0.067)  Michigan (0.049)  Minnesota (0.053)  New Hampshire (0.048)  New Jersey (0.066)  New York (0.081)  Pennsylvania (0.048)  South Carolina (0.048)  Virginia (0.049)  Wisconsin (0.046) | Arkansas (0.086)  Connecticut (0.046)  Indiana (0.046)  Iowa (0.048)  Kansas (0.076)  Kentucky (0.066)  Louisiana (0.055)  Michigan (0.046)  Minnesota (0.036)  Missouri (0.060)  North Carolina (0.065)  Ohio (0.043)  Oklahoma (0.107)  Pennsylvania (0.054)  Texas (0.054)  Virginia (0.060)  Wisconsin (0.053) | Alabama (0.063)  Arkansas (0.062)  Colorado (0.059)  Florida (0.061)  Georgia (0.062)  Kansas (0.066)  Kentucky (0.063)  Missouri (0.064)  Oklahoma (0.063)  South Carolina (0.064)  Tennessee (0.063)  Texas (0.064)  Utah (0.062)  Virginia (0.062)  Washington (0.060)  West Virginia (0.061) |
| Some donor pools are relatively small due to a lack of extensive CDC data on suicide mortality outside of the states listed. | | | | | |
