# Supplementary material for: Can additional funding improve mental health outcomes? Evidence from a synthetic control analysis of California’s millionaire tax
Source: PLoS One. 2022 Jul 27;17(7):e0271063. doi: 10.1371/journal.pone.0271063 (PMC9328510; doi:10.1371/journal.pone.0271063)
Supplement: S5 Table — (DOCX) [file pone.0271063.s005.docx]

| **S5 Table.** **Earmarked Mental Health Tax Effect on Suicide Mortality Among California’s General Population Using Alternative Intervention Years.** | | | | |
| --- | --- | --- | --- | --- |
| Year | Intervention Year | | | |
|  | 2006 |  | 2007 |  |
| 2005 | - |  | - |  |
| 2006 | 0.24 |  | - |  |
| 2007 | -0.05 |  | 0.25 |  |
| 2008 | -0.14 |  | -0.05 |  |
| 2009 | -0.75 |  | -0.13 |  |
| 2010 | -0.70 |  | -0.75 |  |
| 2011 | -0.70 |  | -0.70 |  |
| 2012 | -1.26 | *** | -1.26 | *** |
| 2013 | -1.08 | * | -1.06 | ** |
| 2014 | -1.44 | *** | -1.46 | *** |
| 2015 | -1.75 | ** | -1.77 | ** |
| 2016 | -1.65 | ** | -1.68 | *** |
| 2017 | -2.42 | *** | -2.45 | *** |
| 2018 | -2.02 | *** | -2.05 | *** |
| 2019 | -2.15 | *** | -2.16 | *** |
| RMSPE | 0.5574 |  | 0.5293 |  |
| The effect is the reduction in mortality rate (deaths per 100,000); ** *p* ≤ 0.05; *** *p* ≤ 0.01. | | | | |
