# Supplementary material for: Can additional funding improve mental health outcomes? Evidence from a synthetic control analysis of California’s millionaire tax
Source: PLoS One. 2022 Jul 27;17(7):e0271063. doi: 10.1371/journal.pone.0271063 (PMC9328510; doi:10.1371/journal.pone.0271063)
Supplement: Attachment — Submitted filename: Response to Reviewers.docx [file pone.0271063.s006.docx]

**RESPONSE TO REVIEWERS**

My sincere thanks to each reviewer and the editor for evaluating the initial manuscript. As detailed below, the revision incorporates the feedback where possible alongside grammatical edits. Highlights include:

- The models exclude unemployment as a predictor of suicide mortality because various studies question whether that link exists, and a diagnostic model confirmed the non-relationship. Including it and other predictors worsens model fit.
- Deaths from opioid abuse are not classified as intentional self-harm and thus do not affect suicide mortality rates or the study’s results. While some suicides do, in fact, result from opioid overdoses, there are relatively few (only about 0.09% of all self-inflicted deaths), and they are inconsequential to the study’s results. I also found no strong relationship between opioid prescription rates or overdose rates and suicide. I thus doubt that the opioid crisis is responsible for the synthetic control’s upward trajectory.
- The revision includes an additional robustness check using a new (May 2022) method to correct for errors due to imprecise matching between the treatment unit and donor units. The error-corrected treatment effects are nearly identical to the original effects.
- All SCM graphs have the same vertical scale, which facilitates easier comparisons. The ITSA graph matches that scale.

I believe the revision and my “rebuttals” address the reviewers’ concerns. If not, I am happy to make additional edits and appreciate the opportunity to do so.

**Issue:** Reviewer 1 suggested reduced suicide mortality in California relative to its synthetic control may result from omitted variables, especially the Great Recession and unemployment.

**Response:** Like the reviewer, I initially hypothesized unemployment affected suicide mortality. But I ultimately excluded it for three reasons:

- If there was a strong correspondence between unemployment and suicide mortality, the latter should have declined after 2011 as unemployment fell. That did not transpire. Moreover, California’s unemployment rate exceeded the national average during and after the recession. The state’s suicide rate did not.
- The literature relating unemployment to suicide mortality is weaker than the conventional wisdom dictates. Studies tend to correlate the variables without resolving causation. Meta-analyses that fail to exclude those studies and/or that fail to correct for publication bias amplify the purported link. Studies that address that and other methodological issues show unemployment and income do not correspond to overall suicide mortality in Europe (Andrés 2005) or the United States (Minoiua and Andrés 2008). That may be because the relationship has weakened over time (DeFina and Hannon 2015), has a transient impact specific to subgroups (Abdou et al. 2020; Breuer 2015), or because unemployment can be an individual risk factor but not a general population-level factor (Stack 2020). The root cause may be something else altogether: recent studies find broad, not space-specific phenomena that coincide with periods of high unemployment, such as repeated exposure to bad economic news (Vandoros and Kawachi 2021) and stock market volatility (Wisniewski et al. 2020), are the true culprits.
- Confirming the second point, an unreported fixed-effects model that regresses the unemployment rate and other controls (e.g., per capita income, state ideology, and population) on the suicide mortality rate found no statistically significant effect for unemployment (*p*=0.719) or the other predictors.

I am therefore reluctant to revise the synthetic control model to include the unemployment rate. Nevertheless, I agree with the reviewer that this issue merits attention. The conclusion now includes a brief discussion of the potential unemployment-suicide link, and the text explains why it was excluded from the models.

**Issue:** Reviewer 1 suggested that increased opioid abuse may explain the paper’s results.

**Response:** I had not considered this possibility (thank you for bringing it to my attention!) and looked more closely at the CDC’s mortality data. That data separates the following into distinct categories:

- Deaths from accidental drug overdoses involving drugs that act on the nervous system
- Deaths from intentional drug overdoses involving drugs that act on the nervous system
- Deaths due to “Mental or behavioral disorder due to use of opioids” (F11), including acute intoxication, harmful use, dependence syndrome, and withdrawal

The mortality data used in the study include intentional overdoses only. I exclude deaths caused by an *accidental* overdose (i.e., most deaths related to OUD) and those caused by a “mental or behavioral disorder due to use of opioids,” which are also *not intentional*.

Still, including intentional drug overdoses with other self-inflicted deaths does not bias the study’s findings because they are relatively uncommon. Nearly all suicides in the United States involve firearms, suffocation, or intentional poisoning. The CDC reports 791,532 deaths from intentional self-harm from 1999 through 2019. About 12 percent (97,532) were drug-related. Just 0.09 percent (683) stemmed from an intentional overdose of opioid(s) or other drugs acting on the nervous system. There are so few intentional opioid overdoses each year compared to other intentional, self-inflicted causes that re-running the model without them yields the same result.

I calculated two relevant correlations to resolve further the potential association between opioid use and suicide. Both were low and not suggestive of a relationship. In 2018, for example, the correlation between the opioid prescription rate and the suicide mortality rate was 0.27. In the same year, the correlation between opioid-related inpatient stays (a rough indicator of abuse) and the suicide mortality rate was 0.22. (Note: The underlying data derive from the NIH and the Healthcare Cost and Utilization Project, an Agency for Healthcare Research and Quality initiative.)

I agree with the reviewer that this issue merits attention. The revision notes that drug overdose deaths, from opioids or other substances, do not affect the mortality data.

**Issue:** Reviewer 1 asked about the availability of other mental health-related data, perhaps from Medicaid or private insurance firms.

**Response:** The suggested data sources present several problems. First, Medicaid programs and private health insurance plans differ by state, and data is not comparable. Second, I don’t believe they collect much, if any, suicide mortality data. Third, the enactment of the Affordable Care Act during the study’s period, with concomitant changes to both Medicaid (which states did not implement concurrently) and private health insurance plans, likely means the within-state data are not comparable over time. Fourth, and related to the third, public or private health insurance data on mental health diagnoses over the study’s period may reflect increased acceptability of seeking mental health care, not an actual change in mental health status. It is a morbid topic, but the suicide mortality rate is, at the very least, an objective and consistent longitudinal mental health indicator.

**Issue:** Reviewer 1 expressed curiosity about RMSPE values for different predictor sets.

**Response:** Adding unemployment to the model increases the RMPSE to 1.9417 (up from 0.5904). Adding per-capita income increases the RMSPE, probably because the link between income and suicide is not as strong as I thought (see discussion in manuscript; the literature on this question is inconsistent). Adding both as predictors results in an even worse fit. I also tried citizen ideology (which has some weak relationship to mental health). But it, too, worsens fit. The model with the lowest RMSPE is the model presented in the original paper and the revision. Except for 2001, which appears to be an age-based anomaly in suicide mortality that year, California’s mortality rate and the synthetic control are well matched.

**Issue:** Reviewer 1 noted that donor pool states “are geographically and epidemiologically very different from CA” and points to differences in the OUD rate.

**Response:** Given the small number of intentional drug over-dose deaths that involve opioids, not to mention the low correspondence between opioid prescriptions and abuse and suicide mortality, I believe it is implausible that OUD in some donor states skews the synthetic control.

I am unaware of any research linking geography and suicide mortality in the United States and note that each model’s donor pool is unique. New York, a donor state in nine models, may not “look like” California and is on the east coast. But importantly, more people move to California from New York than any other state. According to CalMatters, the other states with the highest out-migration to California are Illinois, Pennsylvania, Michigan, Ohio, and Washington. Most, like California, are large states with relatively diverse populations. It is not surprising that these same states often appear as donors with proximate age-adjusted suicide mortality. Otherwise, I’m not sure what the reviewer means by “epidemiologically similar.”

**Issue:** Reviewer 2 noted “we still like to see some sensitiveness analysis due to these subjective constraints/exclusion (page 7).” The reviewer suggested “applying the permutation test to evaluate the significance and robustness of the estimations.”

**Response:** I am not sure what the reviewer means by “permutation test.” The original manuscript presented permutation test results. They are also reported in the revision on pages 8-9.

As noted in the summary, the revision includes a new, additional robustness check that rules out any bias due to imprecise matching on predictor variables.

**Issue:** Reviewer 2 observed “[t]here are no details on the modeling approach used and no equations in the paper that would enable us to judge/reduplicate what exactly is being modeled.”

**Response:** I excluded equations because recent tax- and health-related articles in PLOS ONE also did so (e.g., Gunadi et al.’s (2021) assessment of California’s tobacco tax increase and Marinello et al.’s (2021) analysis of San Francisco’s sugar-sweetened beverage tax). I am happy to add details if necessary; perhaps the editor could advise.

**Issue:** Reviewer 2 suggested that the paper show “how much tax money could deter one suicide life in different age/ethnic/gender groups in California.”

**Response:** That is a great idea. But I hesitate to put a “price” on life because the funding doesn’t only prevent suicide; it supports mental health care more generally. I could include a rough estimate (i.e., total funding divided by suicides avoided), but it could mislead readers. I am open to suggestions on this matter.

**References:**

Abdou, Rayayda, Damien Cassells, Jenny Berrill, and Jim Hanly. 2020. “An Empirical Investigation of the Relationship between Business Performance and Suicide in the US.” Social Science & Medicine 264: 113256.

Andrés, Antonio Rodríguez. 2005. “Income Inequality, Unemployment, and Suicide: A Panel Data Analysis of 15 European Countries.” Applied Economics 37(4): 439-451.

Breuer, Christian. 2015. “Unemployment and Suicide Mortality: Evidence from Regional Panel Data in Europe.” Health Economics 24(8): 936-950.

DeFina, Robert, and Lance Hannon. 2015. “The Changing Relationship between Unemployment and Suicide.” Suicide and Life-Threatening Behavior 45(2): 217-229.

Minoiu, Camelia, and Antonio Rodríguez Andrés. 2008. “The Effect of Public Spending on Suicide: Evidence from U.S. State Data.” The Journal of Socio-Economics 37(1): 237-261.

Stack, Steven. 2020. “Contributing Factors to Suicide: Political, Social, Cultural and Economic.” Preventative Medicine 152: 106498.

Vandros, Sotiris, and Ichiro Kawachi. 2021. “Economic Uncertainty and Suicide in the United States.” European Journal of Epidemiology 36(6): 641-647.

Wisniewski, Tomasz Piotr, Brendan John Lambe, and Keshab Shrestha. 2020. “Do Stock Market Fluctuations Affect Suicide Rates?” The Journal of Financial Research 43(4): 737-765.
