# Supplementary material for: Can additional funding improve mental health outcomes? Evidence from a synthetic control analysis of California’s millionaire tax
Source: PLoS One. 2022 Jul 27;17(7):e0271063. doi: 10.1371/journal.pone.0271063 (PMC9328510; doi:10.1371/journal.pone.0271063)
Supplement: Attachment — Submitted filename: Response to Reviewers.docx [file pone.0271063.s007.docx]

**RESPONSE TO REVIEWERS**

My continued thanks to both reviewers and the editor. This second revision incorporates one of the two changes proffered by reviewer #1; the other, which I believe the same reviewer offered simply as a suggestion, is not workable. I am happy to make additional edits and appreciate the opportunity.

**Issue:** Reviewer 1 suggested reporting two sets of results, one with and one without mortality from drug overdoses.

**Response:** The manuscript focuses on intentional self-inflicted deaths from any cause, which may include—but often does not—drug overdoses. As a robustness check, I expanded the definition of mortality to include intentional self-inflicted deaths and accidental drug overdoses, such as from opioids, cocaine, and sedatives. Since the law focuses on mental health, with substance abuse secondary, I think that’s the best way to present the results (intentional deaths first; intentional and some accidental deaths secondary). I did not include all the particulars—there are now five separate robustness checks, far more than other studies published in PLOS One—but the pattern endures: even under the expanded mortality definition, California’s rate grows much more slowly than the synthetic control. As an additional verification, I applied ITSA to the expanded mortality rate data and confirmed the synthetic control result. The manuscript’s text is updated to include a discussion of the additional robustness check. It also notes that ITSA returns the same finding.

Bottom line: Regardless of whether drug overdoses are included, California fared better than the control, and the conclusion is robust to multiple sensitivity checks. The manuscript now makes that clear.

**Issue:** Reviewer 1 suggested using 911 call data.

**Response:** 911 systems in the United States are local. I am unaware of a centralized database of all call data from 1999 for every system in each state. I do not believe requesting that data—if it even exists—will yield a value-add to the current manuscript. There are two additional complicating factors. First, local systems do not operate according to identical protocols for coding calls or referring them. Second, the data—again, if it even exists—is not consistent over the study’s time period, as many systems switched from older technology to E911 (which makes intersystem referrals easier).
